# In This Issue

**Published:** 2003

## CLASSIFICATION OF ALCOHOL USE DISORDERS

Clear, accurate definitions of medical conditions and disorders are important for both research and clinical practice. Treatment studies, human genetics research, and national prevalence estimates of alcoholism all rely on certain sets of criteria to define alcohol abuse and dependence. The most widely used definitions for alcohol use disorders are found in two major classification systems of disease: the Diagnostic and Statistical Manual of Mental Disorders (DSM) of the American Psychiatric Association, and the International Classification of Disease (ICD) of the World Health Organization. Dr. Deborah Hasin provides historical background on the development of the current classification systems and describes the key similarities and differences between DSM–III–R, DSM–IV, and ICD–10. She concludes with a discussion of the reliability and validity of the alcohol dependence and abuse diagnoses. (pp. 5–17)

## METHODOLOGICAL ISSUES IN MEASURING ALCOHOL USE

When conducting surveys to measure a population’s alcohol consumption patterns, researchers must consider numerous methodological issues to obtain valid and meaningful information, writes Dr. Deborah A. Dawson. For example, investigators must determine the most appropriate reference period for the surveys (i.e., whether to assess consumption over the past week, month, or year) or whether to assess overall consumption or consumption for various types of beverages. The choice of questions also influences the accuracy of the data. With the quantity/frequency (QF) approach, researchers inquire only about the overall frequency of alcohol consumption and the typical number of drinks consumed per occasion. The graduated frequency (GF) approach asks respondents how often during the reference period they consumed a certain number of drinks and thus generates more detailed information. The specific definitions of drinking status (e.g., abstainer or drinker) as well as the mode of the interview (e.g., face to face vs. telephone) also can affect the responses and, accordingly, estimates of alcohol consumption. (pp. 18–29)

## TRACKING ALCOHOL CONSUMPTION OVER TIME

Researchers regularly track a nation’s alcohol consumption not only to discern current drinking patterns but also to monitor trends in drinking patterns that might influence prevention or intervention approaches and policy changes. Drs. Thomas K. Greenfield and William C. Kerr review the main approaches to measuring alcohol consumption over time. One method is the use of aggregate measures, which summarize the volume of consumption of many individuals to yield a group total or average consumption. Aggregate measures are used primarily to generate models of alcohol-related outcomes, such as alcohol-related mortality. Investigators also conduct surveys (cross-sectional and longitudinal) to measure people’s consumption either at one point or repeatedly over time. Survey data make it possible to obtain a clearer picture of the dynamic forces that shape a population’s consumption patterns. (pp. 30–38)

## ALCOHOL-RELATED MORBIDITY AND MORTALITY

Alcohol use is a contributing factor in a wide range of negative health consequences, including injuries, disease, and disability. Research on alcohol-related morbidity and mortality examines the effects of overall alcohol consumption as well as varying drinking patterns. As Drs. Jürgen Rehm, Gerhard Gmel, Christopher T. Sempos, and Maurizio Trevisan explain, this epidemiological research indicates that alcohol use increases the risk for both chronic health consequences (e.g., disease) and acute consequences (e.g., traffic crashes). The authors note too that certain patterns of regular, light-to-moderate drinking may have beneficial effects, such as a decrease in the risk of coronary heart disease. The authors discuss the methodology involved in studying alcohol-related morbidity and mortality, including measuring alcohol consumption and outcomes. (pp. 39–51)

## HARMFUL ALCOHOL USE

Misuse of alcohol affects not only the drinker but also his or her family, friends, community, and society as a whole. Drs. Gerhard Gmel and Jürgen Rehm examine research on some of the social harms commonly associated with drinking, including lowered worker productivity and increased rates of accidental injuries, aggressive and violent behavior, and child and spouse abuse. The authors note that although drinking is linked to many of these social harms, the exact association is not clear. The authors describe the methodological flaws that characterize much of the research in this area and specify areas where research can be improved. (pp. 52–62)

## EPIDEMIOLOGY AND CONSEQUENCES OF DRINKING AND DRIVING

Drinking and driving remains a significant public health threat in the United States. Approximately 40 percent of traffic deaths in the United States are alcohol related, and crashes involving alcohol are more likely to result in injuries and deaths than non-alcohol-related crashes. Dr. Ralph Hingson and Mr. Michael Winter review the epidemiology of alcohol-related crashes, injuries, and deaths and the relationship between blood alcohol concentration (BAC), driver impairment, and fatal crashes. The authors also examine characteristics of alcohol-related crashes and fatalities with respect to the driver’s age when he or she first began drinking. Drinking and driving survey data and trends in drinking and driving also are discussed. (pp. 63–78)

## ALCOHOL USE AMONG ADOLESCENTS AND YOUNG ADULTS

Young people in the United States have high rates of alcohol use and often engage in dangerous drinking practices such as binge drinking. Dr. Michael Windle reviews epidemiological data on alcohol use among adolescents, college students, and young adults not in college, as well as the prevalence of health-compromising behaviors such as tobacco use and drinking and driving that often co-occur with alcohol use in this age group. The author explains that the patterns and consequences of drinking experienced by young people often are related to the physical locations or the contexts in which alcohol is consumed. Information on drinking locations and contexts may be especially useful for identifying appropriate targets for effective interventions and social policies. (pp. 79–86)

## ALCOHOL USE AND RELATED PROBLEMS AMONG ETHNIC MINORITIES IN THE UNITED STATES

How do minority groups in the United States differ from one another and from Whites in their drinking patterns and in the prevalence of alcohol-related problems? Drs. Frank H. Galvan and Raul Caetano examine the epidemiology of alcohol use and related problems among the four main U.S. minority groups: Hispanics, African Americans, Asian Americans, and Native Americans. The authors review factors thought to account for these ethnic/racial differences, including social or cultural factors such as drinking norms and attitudes and, in some cases, genetic factors. Understanding ethnic differences in alcohol use patterns and the factors that influence alcohol use can help guide the development of culturally appropriate alcoholism treatment and prevention programs. (pp. 87–94)

## INTERNATIONAL COMPARISONS OF ALCOHOL CONSUMPTION

In recent years, researchers have attempted to compare data on drinking for various countries to further advance theoretical knowledge of the social, cultural, political, or environmental factors that influence drinking behavior. In these cross-national analyses, investigators must consider factors such as differences in drinking cultures (e.g., the role that alcohol plays in everyday life), variations in drink sizes and strengths, and differing methods for measuring consumption. With these considerations in mind, Drs. Kim Bloomfield, Tim Stockwell, and Gerhard Gmel and Ms. Nina Rehn summarize the findings of international research. Although the existing studies already have generated much information about drinking behavior in several countries, numerous questions and methodological concerns remain, necessitating further research. (pp. 95–109)

## ACCIDENTAL ALCOHOL POISONING MORTALITY IN THE UNITED STATES, 1996–1998

This article is the latest in the National Institute on Alcohol Abuse and Alcoholism’s (NIAAA’s) Epidemiological Bulletin series. Drs. Young-Hee Yoon, Frederick S. Stinson, Hsiao-ye Yi, and Mary C. Dufour examine the prevalence and patterns of mortality resulting from accidental alcohol poisoning in the United States. The authors report that, for the years 1996 through 1998, the annual average number of deaths for which alcohol poisoning was the underlying cause was 317, with an age-adjusted death rate of 0.11 per 100,000 population. An average of 1,076 additional deaths included alcohol poisoning as a contributing cause, bringing the total number of deaths with any mention of alcohol poisoning to 1,393 per year (0.49 per 100,000 population). The article also examines how other factors, such as age, gender, level of education attained, marriage, and race/ethnicity, may place some people at greater risk for death from alcohol poisoning. Information gleaned from studies such as this can help in the design of highly targeted intervention and prevention approaches and lead to more informed policy decisions on this important topic. (pp. 110–118)

